# The effect of low intensity pulsed ultrasound on mandibular condylar growth in young adult rats

**DOI:** 10.1016/j.bonr.2021.101122

**Published:** 2021-09-03

**Authors:** Yasamin Hadaegh, Hasan Uludag, Douglas Dederich, Tarek H. El-Bialy

**Affiliations:** a School of Dentistry, University of Alberta, Edmonton, Canada; b Department of Chemical and Materials Engineering, University of Alberta, Edmonton, Canada

**Keywords:** Mandibular deficiency, LIPUS, Adult animals, Gross morphometric analysis, Micro-computed tomography, Histomorphometric analysis

## Abstract

There is a need for more effective methods to enhance mandibular growth in young adults with mandibular deficiency. Previous studies suggest that low intensity pulsed ultrasound (LIPUS) can enhance mandibular growth in growing individuals. This study aimed to evaluate the potential growth changes of the mandible following 4-week LIPUS application in young adult rats. Nineteen **≈**120-day-old female rats were allocated to experimental (*n* = 10) and control (*n* = 9) groups. The animals in the experimental group were treated with LIPUS to their temporomandibular joints (TMJs) bilaterally, 20 min each day for 28 consecutive days. Animals were then euthanized; gross morphological evaluation was performed on 2D photographs and 3D virtual models of hemi-mandibles, and microstructural assessment was done for the mandibular condyle (MC). Evaluation of mineralization and microarchitecture properties of subchondral cancellous bone was performed by micro-computed tomography (μCT) scanning. Qualitative and histomorphometric analysis was done on condylar cartilage and subchondral bone following Alcian Blue/PAS and Goldner's Trichrome staining. Vital flourochrome (calcein green) labeling was also utilized to determine the amount of endochondral bone growth. Gross morphological evaluations showed a slight statistically non-significant increase especially in the main condylar growth direction in the LIPUS group. Moreover, 3D evaluation depicted an enhanced periosteal bone apposition at the site of LIPUS application. Microstructural analysis revealed that LIPUS stimulates both chondrogenesis and osteogenesis and enhances endochondral bone formation in young adult rat MC. Furthermore, the effect of LIPUS on osteogenic cells of subchondral cancellous bone was notable. To conclude, LIPUS can enhance young adult rats' MC residual growth potential.

## Highlights

• Specialized morphometric and microstructural evaluations was performed.
• LIPUS can enhance mandibular condylar residual growth potential in young adult rats.
• Both chondrogenesis and osteogenesis were stimulated.
• Endochondral bone formation and subchondral trabecular bone remodeling was boosted.
• The tissue response decreases as intrinsic maturity in different regions increases.

## Graphical abstract

**Figure:**
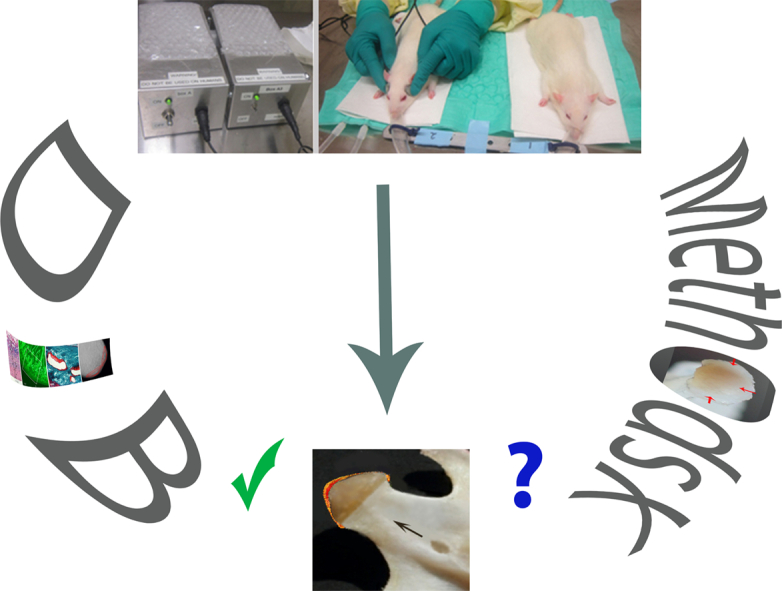

## 1 Introduction

Underdevelopment of the lower jaw is one of the most common deformities of craniofacial region (Pirttiniemi et al., 2009). This malformation not only affects jaw function and esthetic appearance of the face (Proffit, 2013), but also may lead to upper airway obstruction (Flores-Mir et al., 2013).

There are an increasing number of young adult patients with mandibular deficiency who are demanding cost-effective, non-surgical, and high-quality treatment (Sood, 2010). Despite this, surgical interventions for advancing the mandible remain as the preferred choice, but they are costly and risky, and also suffer from limitations and unpredictable stability (Ow and Cheung, 2008, Ow and Cheung, 2009). However, most recently, fixed bite-jumping appliances have been utilized for treating young adult patients with mandibular deficiency (Ruf and Pancherz, 2006; von Bremen et al., 2014; Bock et al., 2016). Mandibular condyle, grows in postnatal life, mainly by endochondral bone growth and has a pivotal role in development of the mandible and orofacial complex (Patil et al., 2012). Continuous forward positioning of the mandible reactivates chondrogenesis in adult condylar cartilage and eventually leads to increased bone formation (Rabie et al., 2004). Available clinical studies have reported favorable skeletal response and improved facial profiles of patients treated with functional appliances. It has been reported that fixed bite-jumping appliances are the appropriate alternative for borderline adult patients with under-developed mandibles (Kabbur et al., 2012; von Bremen et al., 2014).

Evidently compared to growing individuals, skeletal changes by functional appliances in adults are slower and less which is due to lower growth rate and remodeling activity in adult mandibular condyle (Ruf and Pancherz, 2006; Frye et al., 2009). However, it has been reported that by repeated mechanical loading (i.e., stepwise mandibular advancement) this can be enhanced further (Ng et al., 2006; Aras et al., 2017) even in adults (Purkayastha et al., 2008; Chaiyongsirisern et al., 2009; Austin et al., 2010).

LIPUS is an acoustic pressure wave in the form of repeated mechanical loading that causes nanomotions within biological tissues, and triggers intracellular signaling, leading to specific biochemical events (Harrison et al., 2016). LIPUS has been approved by the food and drug administration (FDA) USA for enhancing bone fracture healing for decades (Harrison et al., 2016). LIPUS stimulates chondrogenesis, cartilage hypertrophy, angiogenesis, and osteogenesis which lead to earlier onset and enhanced endochondral bone formation than the natural process (Harrison et al., 2016; Aliabouzar et al., 2018; Kaur et al., 2018; Crossman et al., 2019; Wang et al., 2019).

It has been shown that application of LIPUS on the TMJ region enhances condylar and mandibular growth in growing species especially under continuous mandibular advancement (El-Bialy et al., 2003, El-Bialy et al., 2006, El-Bialy et al., 2010; Oyonarte et al., 2009, Oyonarte et al., 2013; Khan et al., 2013; Kaur et al., 2014, Kaur et al., 2017; Sasaki et al., 2016; Maurya et al., 2019). However, such a therapeutic efficacy in adults has yet to be evaluated. The aim of the present work was to examine potential morphological changes of the mandible focusing on the condylar process and to depict its detailed microstructural alterations following LIPUS therapy in young adult rats.

## 2 Materials and methods

### 2.1 Animal care, experimental design and LIPUS application

This experiment was approved by the Animal Care and Use Committee for Health Sciences, University of Alberta, Canada (AUP: 000000381-REN1). Sample size calculation was performed using data derived from previous similar studies on non-growing rat species for various parameters based on the following formula (Cleophas and Zwinderman, 2012):

n=2PooledSDmean difference2×z1−α2+z1−β2

To yield a power of 0.8 with a significance level of 0.05 a sample size for each group, considering 20% possible acceptable loss, was calculated to be 9. Nineteen ≈ 110-day old female Sprague Dawley (SD) rats were received from Charles River Laboratories, WILM, MA, USA. This age of SD rats falls in the young adult (twenties) stage for human beings (Losken et al., 1992). The mean weight of the animals was 347.30 g + 14.41 standard deviation (range 319.2–374.5 g) at the beginning of the study. Animals were housed in pairs per cage under standardized conditions. The rats were acclimatized for 10 days and based on their average weight during this period they were harmoniously allocated to experimental (*n* = 10) and control (*n* = 9) groups. In this study, we utilized the clinically approved setting for LIPUS application (Harrison et al., 2016). LIPUS was applied using a custom-built ultrasound device (Smile Sonica Inc., Edmonton, AB, Canada). The LIPUS device generates ultrasound pulses of 1.5 MHz with a duration of 200 microseconds with a repetition rate of 1 KHz. The ultrasound transducer has an emitting area of 1.1 cm^2^ and it generates a temporal average ultrasound intensity of 30 mW/cm^2^ ± 10% of the transducer's surface area. This setting also has been used in previous studies on growth stimulation of the mandible (El-Bialy et al., 2003, El-Bialy et al., 2006, El-Bialy et al., 2010; Oyonarte et al., 2009, Oyonarte et al., 2013; Khan et al., 2013; Kaur et al., 2014, Kaur et al., 2017; Sasaki et al., 2016; Maurya et al., 2019). The TMJ area of both sides of the experimental groups was exposed to LIPUS daily for 20 min in a single application for 4 weeks. Sonication was performed on the cheek area following shaving and using coupling gel (National Therapy Products Inc. Woodbridge, ON, Canada). During the 20-minute LIPUS application, rats were under general anesthesia using Isoflurane (2.5% with 100% oxygen; Pharmaceutical partners of Canada Inc. Richmond Hill, ON, CA). Control animals were also anesthetized for the same period daily to exclude possible effect of anesthesia on general body conditions. The weight of the animals was monitored during the experiment.

All the rats were euthanized by the end of the experiment (after 4 weeks) via intraperitoneal injection of 0.5 ml Euthansol (Vibrac Corporation, Fort Worth, TX, USA) following inhalation anesthesia except for one control rat which died on the 11th day of the experiment. The animals' heads were carefully dissected along the middle sagittal plane. Hemi-mandibles were harvested, cleaned and fixed in a 10% formalin solution (Sigma Aldrich, St Louis, MO, USA) for 48 h at room temperature. At this step coding has been performed to address blinding in subsequent assessments.

### 2.2 Gross morphological evaluation

#### 2.2.1 Morphometric measurements on 2D photographs

On digital photographs of rat hemi-mandibles using AutoCAD software, anatomical landmarks were determined and 16 linear and one angular measurements were performed based on a modified methodology utilized in previous studies (Xiong et al., 2004; Taira et al., 2009). Details are available in …the paper prepared for “MethodsX”.

#### 2.2.2 Linear measurements on 3D virtual models

Two linear measurements, the distances from the midpoint of mandibular foramen and the posterior-inferior point of attachment of digastric muscle to the posterosuperior point of the condyle respectively condylar process length and mandibular length, were also performed using Geomagic-Qualify software on 3D virtual models produced from μCT reconstructed images of the hemi-mandibles. Details can be found in…the paper prepared for “MethodsX”.

#### 2.2.3 3D visualization

To objectively visualize potential morphological differences between experimental and control groups, a modified combination of two methods previously reported in the literature [Mavropoulos et al., 2004; Reynolds et al., 2011] was adopted. Left and right average 3D virtual hemi-mandibles representative of control and LIPUS groups were produced and compared in a form of 3D-deviation map. Detailed method is in the paper prepared for “MethodsX”.

### 2.3 Microscopic histologic evaluation

In this experiment, the left and right mandibular condyles were respectively used for undecalcified and decalcified sections. Sectioning of the condyles was performed at mid sagittal plane.

#### 2.3.1 Alcian blue-PAS staining

This technique was used in the present study to identify various fibrocartilage cell layers (Xiong et al., 2005a), also to discern newly formed bone and calcifying cartilage from mature bone (Rabie et al., 2004). Slide preparation and staining protocol were performed based on the method suggested by Rabie et al. (2003). Histomorphometric evaluations were performed in the middle and posterior regions of the right condyles. More details about the method is described in …the paper prepared for “Data in Brief”.

#### 2.3.2 In vivo flourochrome labeling

To determine the potential actual endochondral bone growth (Purkayastha et al., 2008), 7 and 28 days following the start of the treatment a solution of calcein green was injected into all the rats. The injection was performed subcutaneously using 1 ml syringe with 26 G1/2 precision glide needle (BD, Fanklin, NJ, USA). The calcein green solution was prepared immediately before injection at the dosage of 10 mg/kg by solving a 1- to 1.24 ml of 3 g/l calcein green (C0875-5G, Sigma Aldrich, St Louis, MO, USA) in NaHCO3 2%. The label interval (the time interval between the flourochrome administrations) was 21 days, one to two sigma periods, which is suggested as the most appropriate to investigate directed bone formation (Gaalen et al., 2010). A longer time was avoided to decrease the chances for label escape (Frost, 1969). Vital staining technique and slide preparation were the same as the study by Suzuki et al. (2004). The amount of endochondral bone growth was evaluated in the middle region of the left condyles. More details about the method is described in the paper prepared for “Data in Brief”.

#### 2.3.3 Goldner's trichrome staining for osteoid

This technique was utilized in the present study to determine whether LIPUS application has influence on osteoblast activity and hence osteoid secretion which forms the primary non-mineralized matrix of the bone, in cancellous bone subjacent to condylar cartilage. Staining was performed following the method described by Bemenderfer et al., 2014. The evaluation of osteoid formation was performed in the middle and posterior regions of the left condyles. More details about the method is described in…the paper prepared for “Data in Brief”.

### 2.4 μCT evaluation

To evaluate the effect of LIPUS on bone remodeling activity and bone formation in subchondral cancellous bone, high resolution micro X-ray computed tomography was performed; so that alterations in bone microarchitecture and degree of mineralization was analyzed directly in 3 dimensions. Densitometric and morphometric bone parameters of cancellous bone were evaluated in the middle and posterior regions of the condyle for both left and right hemi-mandibles. More details about the method is described in the paper prepared for “data in brief”.

### 2.5 Statistical analysis

For comparison of the groups on the amount of endochondral bone growth, considering two independent samples and the fact that equality of variance and normality were met, independent sample *t*-test was used. For the rest of measured variables, to compare the groups when compensating for correlation between the outcomes, considering either laterality (left and right hemi mandibles) and/or position (middle and posterior), generalized estimating equation (GEE) was used to analyze the data. To perform two-by-two comparison when considering the multiple comparisons, Bonferroni method was employed. All statistical analysis was performed by SPSS (version 21.0, IBM Co., Chicago, IL, USA). A *P*-value less than 0.05 was considered statistically significant. More details about the statistical analysis and data is available in the paper prepared for “MethodsX” and “Data in Brief”.

## 3 Results

### 3.1 Body weight

The body weight of both experimental and control animals was increased during the acclimatization period and then reduced about 5% following the start of the experiment and remained relatively unchanged until the end of the experiment similarly for both groups.

### 3.2 Gross morphological observations

#### 3.2.1 Morphometric observations on 2D Photographs

**Fig. 1:**
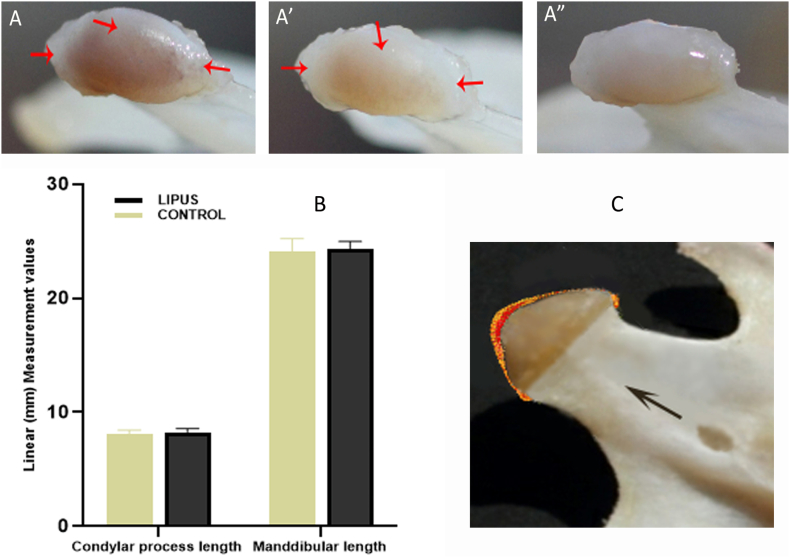
Morphometric observations on the 2D photographs: A, A', A") The condylar cartilaginous cap from LIPUS group with 3 different appearances. Red arrows point to the thicker and more translucent areas. B) The bar chart compares the control and treatment groups for condylar process length and mandibular length (Mean ± SD). C) Picturing of the potential regional changes of the mandibular condyle after LIPUS treatment.

Generally, the adult mandibular condyle is dark and more like a bone due to the very thin covering cartilage. In the present study, the surface of rats' mandibular condyle in both groups was not constantly dark or translucent (Fig. 1-A and A'). However, three pairs of the condyles in the LIPUS group had translucent, thick and large cartilage (Fig. 1-A") and such an appearance was absent in the control group. In addition, it is important to note that in the experimental group, the most top area of the condylar head in almost all samples, also the posterior and anterior end of the cartilaginous cap in some condyles were thick and translucent (Fig. 1-Red arrows). This may indicate enlarged cartilage layer at these regions as evidence of cartilage formation on the condyle surface.

Following the experimental period, there was no statistically significant difference between the groups for all the linear measurements. Nevertheless, in the LIPUS group, the most posterosuperior point of the condyle was in a more posterosuperior location, and condylar process length and mandibular length, which are at the main direction of condylar and mandibular growth (upward, backward), showed higher values. This may suggest a slight growth of the condylar cartilage and/or bone in the top (middle) area of the condylar head, following LIPUS therapy (Fig. 1-B and C). In addition, the observed changes, displayed more of an anterior inferior location of the most anterior superior points of the condyle, which may depict a potential progression of the cartilaginous cap in the perichondrial-periosteal junction consequent to LIPUS treatment (Fig. 1-C). More details of the morphometric analysis can be find in the paper prepared for “MethodsX”.

#### 3.2.2 Morphometric observations on 3D virtual models

**Fig. 2:**
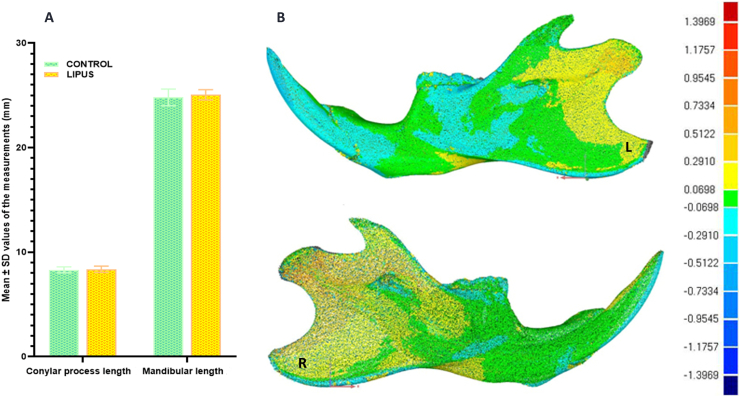
Morphometric observations on the 3D virtual models: A) The bar graph compares the linear measurements in the main direction of condylar and mandibular growth on the 3D virtual models of hemi-mandibles between the control and treatment groups. B) Lateral view of the 3D deviation maps which was the side of LIPUS application. The color map (legend to the right) shows the magnitude of change in millimeters with dark red indicative of maximum positive change (bone apposition), dark blue maximum negative change (bone resorption) and green almost no change.

The linear analyses of 3D virtual hemi-mandibles showed that there was no statistically significant difference in the measurements of the condylar process and mandibular length; however, these measurements were again more in the LIPUS group in comparison to the control group (Fig. 2-A). Fig. 2-B is the lateral view of the left and right virtual models of the 3D deviation map. The orange-yellow areas which can be seen in the 3D deviation maps of both left and right hemi-mandibles may suggest acceleration in periosteal bone apposition in the area of LIPUS application and its vicinity. More details of the morphometric analysis can be find in the paper prepared for “MethodsX”.

### 3.3 Histological and histomorphometric observations

#### 3.3.1 Condylar cartilage

**Fig. 3:**
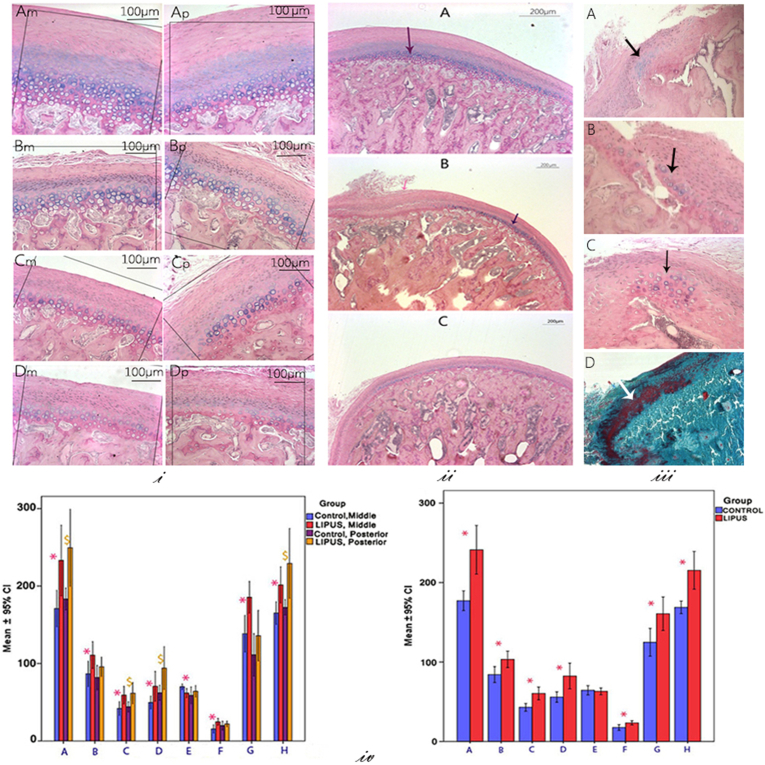
Qualitative and quantitative histological observations on Alcian Blue/PAS stained samples: i-Varied responses of the MC tissue to LIPUS stimuli: 3 animals showed a visually significant thicker fibrocartilage (A), 5 animals showed a moderate increase (B), and 2 showed almost similar characteristics (C) to that of the control (dominant feature) (D); m: middle region; p: posterior region. ii- Region specific response of MC tissue to LIPUS stimuli: Note the increment in the thickness of the chondroblastic layer at the middle region of the condyle (dark violet arrow) (A & B); also the thicker fibrous layer at the posterior region of the condyle (the pink arrow) in the LIPUS group (B) in comparison to the Control (C). iii- Potential emergence or augmentation of the chondroblastic layer in the posterior (A, C, D) and anterior (B) ends of the cartilaginous cap (arrows); D: Goldner's Trichrome staining. iv-Histomorphometric analysis diagram: A: Total fibrocartilage thickness (μm), B: Chondroblastic layers thickness (μm), C: Proliferative layer thickness (μm), D: Fibrous layer thickness (μm), E: BV/TV (%), F: Remnants of calcifying cartilage + newly formed bone area/total bone area (%) G: Cell population in Chondroblastic layer, H: Cell population in Proliferative layer. Left: Region specific comparison of groups (*p < 0.05 middle); (^$^p < 0.05 posterior); Right: Comparison of groups without considering different regions *p < 0.05.

In general, the adult condylar cartilage was thinner compared to that of rapidly growing animals (Jiao et al., 2010). The hypertrophic layer was absent from the anterior region of the condyle of almost all animals and posterior regions of some sections. Hence, the majority of our evaluations were restricted to middle and posterior areas. Even though MC tissue response in the experimental animals varied, the LIPUS group in comparison to the control group showed visually thicker fibrocartilage and increased cellularity in prechondroblastic (proliferative) and chondroblastic (maturation and hypertrophic) layers (Fig. 3-i). In experimental animals, there was increased thickness and cellularity of the chondroblastic layer at the middle region of the condyles (Fig. 3-ii). Moreover, LIPUS treated condyles showed more pronounced positive staining for Alcian Blue especially within the pericellular matrix of chondroblastic region, which is consistent with increased proteoglycan synthesis. Further, chondroblasts were more hypertrophic in the LIPUS group compared to that of the control (Fig. 3-i and ii). Four of the experimental animals showed augmentation of the chondroblastic layer at the anterior and posterior ends of the cartilaginous cap, which may suggest a higher potential of growth or response to LIPUS in these regions (Fig. 3-iii) (Manson, 1968).

Histomorphometric analysis showed that LIPUS group exhibited statistically significant increase in the thickness of the total fibrocartilage layer compared to the control group in both middle and posterior regions. Although the increase in thickness happened in all the layers in the posterior region, it did not reach a statistically significant level in the chondroblastic layer. In contrast, the prechondroblastic and fibrous layers in the posterior region showed respectively similar and larger augmentation compared to the middle region. Likewise, a statistically significant increase of cell number was observed in the prechondroblastic layer in both evaluated regions while in chondroblastic layer it was observed only in the middle region. When the analysis was performed for both regions together (Total), all the above-mentioned parameters showed significantly higher values for the LIPUS group compared to that of the control (Fig. 3-iv).

#### 3.3.2 Subchondral cancellous bone

In the middle region, bone volume fraction (bone area of subchondral trabecular bone/tissue area) was significantly lower and the percentage of remnants of calcifying cartilage and newly formed bone area/bone area were significantly higher in the experimental group compared to that of the control. However, in the posterior region the values for both parameters in the experimental group were slightly higher than that of the control, but not to a statistically significant degree (Fig. 3-iv). Combining the results for both regions together revealed significantly higher values for the percentage of remnants of calcifying cartilage and newly formed bone areas/bone area in the LIPUS group when comparing to the control group (Fig. 3-iv).

**Fig. 4:**
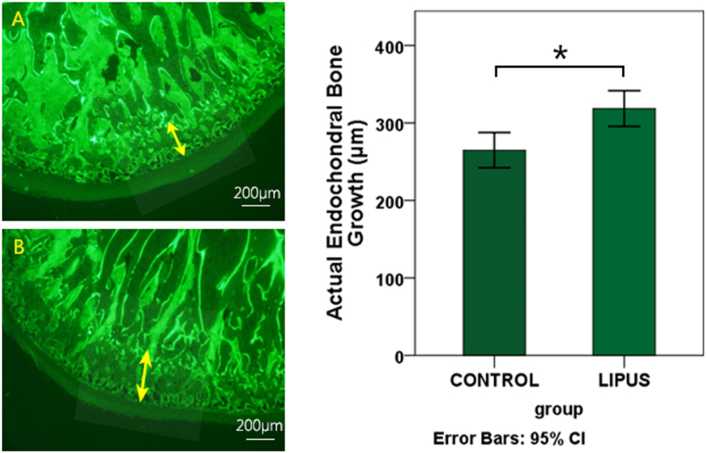
Change in the amount of actual endochondral bone growth in the MCs, determine by vital flourochrome labeling: The distance between two calcein labels echoes the amount of endochondral bone growth during three weeks of the experimental period in the control (A) and LIPUS (B) groups. The bar chart demonstrates the same comparison *p < 0.01.

**Fig. 5:**
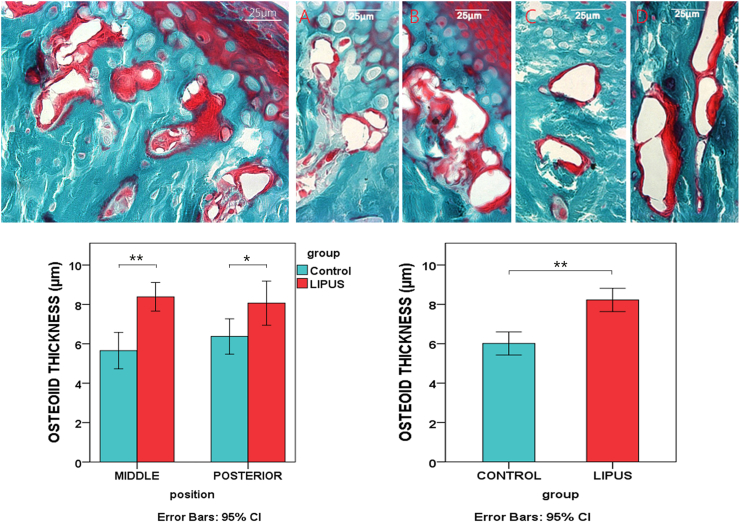
Changes in the osteoid structure of subchondral cancellous bone in the MC on Goldner's Trichrome stained samples: Upper) Left: excessive osteoid formation subjacent to cartilage in the middle region of the condyle from the LIPUS group. Right: comparison of the osteoid formation in the control (A: middle region, C: posterior region) and LIPUS (B: middle region, D: posterior region) groups. Lower) The comparison of the osteoid thickness (μm) in the experimental and control groups; region specific comparison (Left), total comparison without considering region (Right) * *p* < 0.01, **p < 0.001.

The amount of actual endochondral bone growth was significantly higher in the LIPUS group than that in the control group (Fig. 4). Also, in the LIPUS group, osteoid formation was greater and in some locations in a non-organized fashion in subchondral cancellous bone compared to the center (far from cartilage) of the condyle (Fig. 5). Osteoid thickness in trabecular bone subjacent to cartilage bone junction was significantly higher in the LIPUS group than in the control group, especially in the middle region. It should be noted that, varied tissue responses among experimental animals were also present at the bone level.

### 3.4 μCT observations

**Fig. 6:**
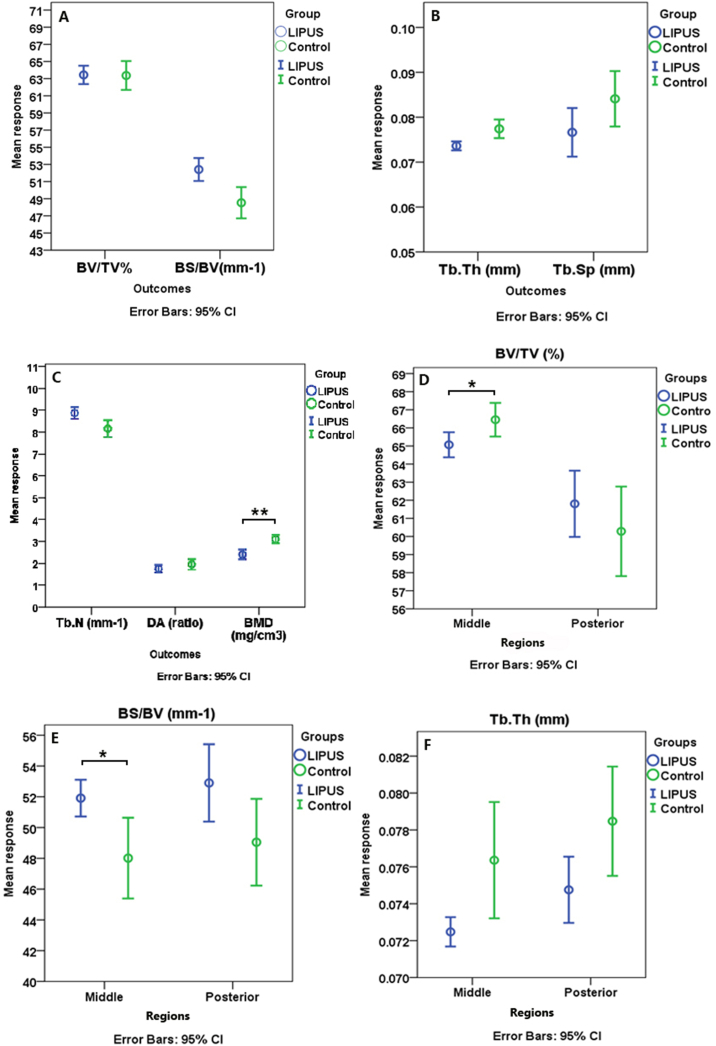

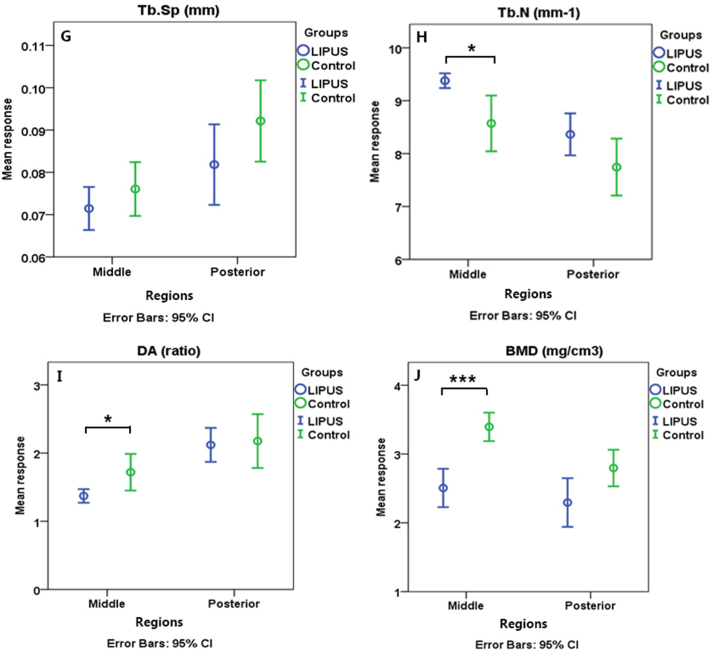
μCT observations: The comparison of densitometric and morphometric parameters of the condylar subchondral bone in the middle and posterior regions between the control and LIPUS groups; A–C: comparing the groups without considering regions. D–J: Region specific comparison of the groups *p < 0.05, **p < 0.01, ***p < 0.001.

μCT analysis showed no statistically significant difference in the subchondral trabecular bone in the middle and posterior regions together between groups. However, Bone Mineral Density (BMD (mg/cm3)) showed significantly lower values for the LIPUS group compared to that of the control. Although statistically nonsignificant, in experimental group, higher values of Bone Specific Surface (Bone Surface to volume ratio) (BS/BV(mm-1)) and Trabecular Number (Tb.N (mm-1)) along with lower Trabecular Thickness (Tb.Th (mm)) and Degree of Anisotropy (DA (ratio)) values comparing to the control were notable (Fig. 6(A–C)).

In the middle region, LIPUS group in comparison to the control, showed significantly higher BS/BV and Tb.N, significantly lower DA, BMD, Bone Volume Fraction (Bone Volume/Tissue Volume) (BV/TV (%)) and marginally significantly lower Tb. Th values. Trabecular Separation (Tb. Sp (mm)), however, remained almost unchanged which might be due to the higher impact of therapy on trabecular number rather than thickness. Apart from BV/TV, for all the above-mentioned parameters a similar trend was observed in the posterior region. However, none of the differences between LIPUS and control groups for any of the outcome variables reached a statistically significant level in this region (Fig. 6(D–J)).

## 4 Discussion

In the present study, our results from gross morphometric evaluations demonstrated that LIPUS application for 28 days, in young adult rats corresponding to a human equivalent period of 32 months (Pancherz and Ruf, 2008), is not sufficient for statistically significant increase in mandibular or condylar size. Comparing the average 3D virtual hemi-mandibles from experimental and control groups in the form of a 3D deviation map was suggestive of enhanced periosteal bone apposition at the site of LIPUS application, which resembles the effect of LIPUS on long bones (Naruse et al., 2009; Perry et al., 2009).

According to our histomorphometric observations in the cartilage level, even though MC tissue response in young adult rats was varied, the LIPUS group in comparison to the control group in general showed statistically significant thicker fibrocartilage, increased cellularity and boosted proteoglycan synthesis. Similar histologic changes have been reported by previous studies in which the effect of LIPUS has been evaluated on MC growth modification in growing (El-Bialy et al., 2003; Oyonarte et al., 2009, Oyonarte et al., 2013) as well as late adolescent (Kaur et al., 2014) rodents. The stimulatory effect of LIPUS on fibroblast proliferation, activity, and collagen synthesis has been documented in previous studies (De Oliveria Perrucini et al., 2020). Moreover, in vitro and in vivo studies have depicted the chondrogenic potential of the LIPUS even on adult cells obtained from aged patients (Korstjens et al., 2008; Kanaguchi Arita et al., 2018).

Interestingly, in the present study the cell population as well as the thickness of chondroblastic layer were more in the middle region in the control group compared to the posterior region and their increment as a result of LIPUS application were statistically significant in this region but not in the posterior area. Hypertrophic chondrocytes appear during the maturation of cartilage and are at the terminal stage of differentiation, but are still metabolically active for the maintenance of cellular morphology and protein synthesis (Hickok et al., 1998). Therefore, hypertrophic chondrocytes should be capable of responding to physical stimuli such as LIPUS. In the present experiment, the increased cell population and thickness of matured and hypertrophic cell layer as well as more hypertrophic cells and increased proteoglycan synthesis in the LIPUS group compared to that of the control agrees with this statement as well as previous studies (Azuma et al., 2001; Zhang et al., 2002). It is generally agreed that the hypertrophy of chondrocytes is a crucial step in endochondral bone formation. While the above-mentioned effect might be transient, it could be the basis for the acceleration of bone formation by LIPUS. In fact, hypertrophic chondrocytes produce calcified cartilaginous matrix and angiogenic stimulators, thus becoming a target for capillary invasion and neovascularization which marks the onset of ossification (Alini et al., 1996) and it is well documented that LIPUS can accelerate endochondral ossification through neovascularization (Harrison et al., 2016; Crossman et al., 2019).

Subchondral cancellous bone in adults is composed of dense and compact trabecular bone with high bone mineral density, while the growing condyle, in which endochondral bone formation is actively progressing, has a low BMD, BV/TV (small bone area), trabecular thickness, and more trabecular number with larger trabecular surface area (Jiao et al., 2010). Moreover, the degree of anisotropy in woven bone, which forms under conditions of rapid turnover like normal growth, is low while by maturation it increases (Daegling and Grine, 2007). Based on our histomorphometric evaluations of the condyles, the percentage of bone area of subchondral trabecular bone/tissue area was significantly lower and the percentage of remnants of calcifying cartilage and newly formed bone area/bone area, in the middle region, was significantly higher in the experimental group compared to that of the control. Furthermore, μCT analysis showed significantly higher BS/BV and Tb.N along with marginally significant and significant lower values for Tb. Th and BV/TV, respectively which displays larger trabecular surface area (Renders et al., 2007) and significantly lower DA (characteristic of woven bone) and BMD. However, in the posterior region none of the bone micro structural and densitometric parameters showed significant changes while a tendency was present. Thus, the abovementioned changes in the subchondral cancellous layer which were more prominent in the middle region of the condyle could be indicative of increased bone remodeling and active bone formation in this area.

Hence, seemingly MC tissue response to LIPUS stimuli is different to that of long bone's epiphysis (Naruse et al., 2009). The mandibular condylar cartilage (MCC), is a secondary cartilage and in contrast to primary cartilages originates from alkaline phosphatise-positive cells of periosteum (Shibata et al., 2000). These relatively undifferentiated cells are responsible for the growth of the MCC by their proliferation and differentiation (Carlson et al., 1980). In vitro and in vivo studies in young tissues as well as adult bone demonstrated the stimulatory effect of LIPUS on periosteum and induction of bone growth (Naruse et al., 2009; Perry et al., 2009; Maung et al., 2021). In addition, the MCC is covered by a fully developed mesenchymal tissue layer, in which prechondroblasts are not surrounded by an intracellular matrix to isolate them from local factors (Voudouris and Kuftinec, 2000; Delatte et al., 2005).

Another notable point is that, in contrast to the growth plate fusion in long bones which involves the depletion of the finite progenitor cells in the resting zone, the MCC does not fuse and it appears that growth can continue after appendicular skeletal maturation (Robinson et al., 2017). However, it is thought that by transition from adolescence to adult the role of the condyle will change from a growth site to an articular function. During the transformation, the cartilaginous matrices slowly disappear from the anterior region (Mizoguchi et al., 1993) and posterior slope (Takahashi et al., 1996) of the condyle, and decrease in cellularity occurs last at the top (the middle region) of the condyle (Luder, 1998); Such that a recent study in mice showed normal depletion of progenitor cells in the top of the condyle does not occur until after 9 months (Robinson et al., 2017). likewise, endochondral ossification ceases and a compact subchondral bone plate forms gradually from the periphery of the condyle (Mizoguchi et al., 1993; Luder, 1998; Lei et al., 2013). Thus, the middle region of the condyle maintains zones of unmineralized growth cartilage and undifferentiated mesenchyme, into later stages (Paulsen et al., 1999), which could be due to contribution of this region to resist articulating loads caused by mastication (Mizoguchi et al., 1993). Hence, it can be accepted that in our study the top part of the rat's condyle, with less mature tissue, was more sensitive to LIPUS stimuli and increase in cartilage and bone formation led to translucency and the subtle growth in this region. Moreover, cartilage-bone junction is also more sensitive to external stimuli (Manson, 1968) and is considered to be affected by the tensional force generated by mandibular movement (Weijs, 1973). This may explain the potential progression of the cartilaginous cap in the condyle's perichondrial-periosteal junction have seen in response to LIPUS treatment.

It has been reported that mechanical stimuli produced by forward mandibular positioning (continuous bite-jumping) in the same species, gender and age as in the present study, led to a statistically significant increase in the length of condylar process as well as mandibular length over 30 days (Xiong et al., 2004). The increment in mesenchymal cell population in proliferative layer and thickness of different cartilaginous layers as a result of this therapy were greater than that following LIPUS application in our study; 2 to 6 versus 1.2 to 1.4-fold of the control group (Xiong et al., 2005a). Additionally, the magnitude of changes in subchondral bone microstructure parameters following LIPUS application were less compared to similar evaluations in the posterior area of the condyle in the same population subsequent to continuous bite-jumping. However, the trend was relatively similar (Xiong et al., 2005b). In the present study the percentage of remnants of calcifying cartilage with newly deposited bone areas/bone area showed a 59% increase in the LIPUS group compared to that of the control. In comparison, the amount of increase in new bone formation was 318.91% as a result of forward mandibular positioning (Rabie et al., 2004). We might have missed the peak of changes by the end of the experiment; nevertheless, such a lower tissue response most possibly infers that the mechanical stimuli produced by LIPUS and its subsequent effect is much lower than that of continuous bite jumping and we have yet to reach to that stage. Thus, we may presume that if the applied loading stimulus by functional therapy is enough to induce a maximal chondrogenic and osteogenic response for MCC growth, then it would be unlikely that the slight effects of the ultrasound regimen in the current study would increase the response.

In a widely accepted bone growth evaluation using vital calcein green labeling, which determines if the histological changes are not temporary, our results suggested slightly but significantly higher actual endochondral bone growth in the LIPUS group compared to that of the control. In a previous study (Suzuki et al., 2004) in which Insulin Like Growth Factor 1 (IGF-1) was injected 3 times into the condyle of adult male rats, the treatment led to an increment in total fibrocartilage layer which was less than three times higher than the effect of LIPUS on fibrocartilage thickness in the present study. However, a decline in the percentage of bone area in the subchondral cancellous bone as a result of this treatment was only 4% more than that of the present study and the amount of endochondral bone growth merely 12 μm higher. This could be relevant as the IGF-1 was injected into the condyle and should have a more profound effect on the cartilage with merely an indirect effect on the subchondral cancellous bone. In contrast, whereas LIPUS has a lower stimulatory effect on both structures, it might have a direct effect on osteogenic cells of subchondral cancellous bone as well.

Osteoblasts begin the process of forming bone tissue by secreting the osteoid. Under conditions of rapid bone turnover and growth, osteoblasts produce osteoid rapidly and in a disorganized fashion so woven bone is formed (Testori et al., 2013). In this study, LIPUS treatment resulted in a significant increase in osteoid thickness in subchondral cancellous bone subjacent to cartilage bone junction as a result of increased osteoid apposition and suggests an anabolic effect of LIPUS on the activity of osteoblasts. The significant increment in osteoid thickness happened in both middle and posterior areas but was more prominent in the middle region. This might suggest that rather than an indirect effect, through a stimulatory effect on hypertrophic cells, LIPUS has a direct effect on osteogenic cells of subchondral cancellous bone, which also can be supported by previous studies (Naruse et al., 2003).

Among the available noninvasive treatments for adults with underdevelopment of the lower jaw, stepwise mandibular advancement using fixed bite jumping appliances clinically seems to be most promising (Purkayastha et al., 2008; Chaiyongsirisern et al., 2009). In this treatment, each step should be elongated for 6 months to let the newly formed bone (with type III collagenous matrix) mature to a more stable type I collagenous matrix (Ow and Cheung, 2008). It is suggested that LIPUS can enhance bone maturation and may accelerate type I collagen maturation (Saito et al., 2004; Hsu et al., 2011). It is also noteworthy that the most significant effect of LIPUS on fracture repair is to accelerate and shorten the process of converting soft, cartilaginous callus to hard, mineralized callus and to increase the mechanical stability of the healing fracture (Harrison et al., 2016). Thus, based on these reports as well as our abovementioned findings, future research that includes concurrent LIPUS application and fixed functional appliances in a stepwise manner may decrease the treatment time in each step and may provide stability of the treatment outcome.

## 5 Conclusion

The present study showed that LIPUS can stimulate both chondrogenesis and osteogenesis in young adult rat MC; also, can enhance endochondral bone formation and subchondral trabecular bone remodeling and this response is region specific. The middle (top) region maintains growth cartilaginous appearance into later stages and so is probably more responsive to LIPUS stimuli. Thus, LIPUS may enhance the residual growth potential of the adult condyle rather than the reactivation of MC growth. On the other side, LIPUS showed a viable direct effect on osteogenic cells of subchondral cancellous bone in adult condyles. Comparing our results to that of the previous studies, it is less likely that these biological effects increase the MC growth via fixed bite jumping appliances in young adults; however, it may decrease the treatment time for the potential stable results.

## CRediT authorship contribution statement

Yasamin Hadaegh performed all animal experiments, lab work, data analyses and wrote drafts of the manuscript.

Hasan Uludag provided supervision on the study design, data interpretation and manuscript revision.

Dauglas Dederich provided supervision on the study design, data interpretation and manuscript revision.

Tarek H. El-Bialy was the main supervisor for the first author and provided all scientific support to the first author starting from study design, experiment performance, data analysis and finish manuscript as well as is the corresponding author of this manuscript.

## Declaration of competing interest

The authors declare no conflict of interest.
