# Corrigendum to “The effect of low intensity pulsed ultrasound on mandibular condylar growth in young adult rats” [Bone Rep. 15 (2021) 101122 (December)]

**DOI:** 10.1016/j.bonr.2022.101652

**Published:** 2023-01-02

**Authors:** Yasamin Hadaegh, Hasan Uludag, Douglas Dederich, Tarek H. El-Bialy

**Affiliations:** a School of Dentistry, University of Alberta, Edmonton, Canada; b Department of Chemical and Materials Engineering, University of Alberta, Edmonton, Canada

To provide further clarification for the printed version of the above article, authors would like to inform:

1. More details about the methods, analysis and the data related to the histomorphometric, micro computed tomographic and morphometric evaluations can be find in the two separate articles published by Data in Brief journal, https://doi.org/10.1016/j.dib.2022.108185 and https://doi.org/10.1016/j.dib.2022.108664 respectively. Accordingly, the updated graphical abstract is represented here:

**Figure:**
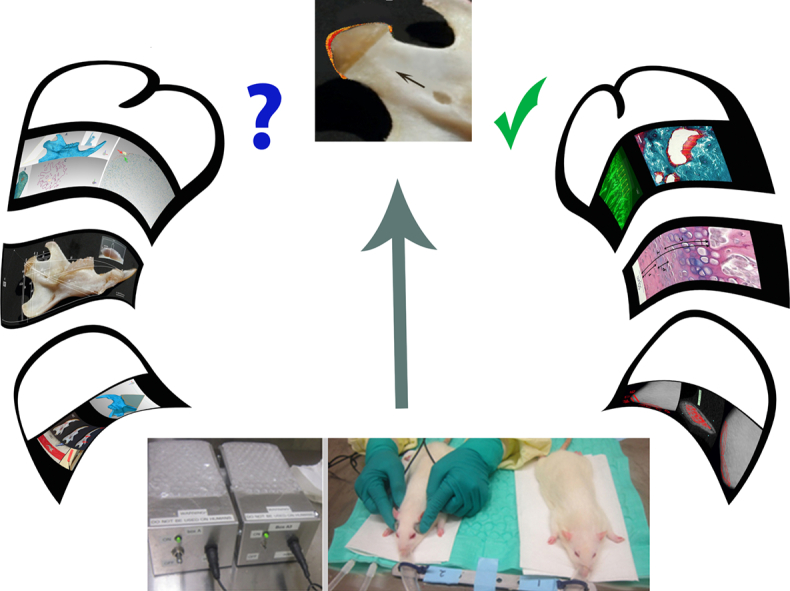

2. Detailed method on producing the 3D deviation map (Part 2-2-3) is as follows:
  A. Averaging the hemi mandibles from each group: Initially, STL files of all hemi-mandibles were optimized using the built-in mesh-doctor feature. Then, superimposition using best fit alignment algorithm was done. Lastly, averaging using a built in average polygon tool in Geomagic Qualify was performed. The result was four 3D virtual models representative of left and right hemi-mandibles from control and LIPUS groups.
  B. Producing 3D Deviation map: For computing 3D deviation map, control and LIPUS hemi-mandibles were used as “reference” and “float” objects respectively.

The authors would like to apologise for any inconvenience caused.

